# Effects of temperature and resource level on interspecific interactions in two species of Odonata larvae

**DOI:** 10.1002/ece3.11502

**Published:** 2024-06-13

**Authors:** Lisa Stahl, Frank Johansson

**Affiliations:** ^1^ Department of Ecology and Genetics, Animal Ecology Program Uppsala University Uppsala Sweden

**Keywords:** growth rate, interspecific interaction, Odonata, resource, temperature

## Abstract

Identifying how temperature and food resources affect interactions between species is important for understanding how climate change will shape community structure in the future. Here, we tested how temperature and resource density affect survival and growth in the larval stage of two coexisting odonates: the damselfly *Lestes sponsa* and the dragonfly *Sympetrum vulgatum*. We performed a laboratory experiment at two temperatures (21 and 24°C) with two resource densities. We estimated the timing of egg hatching of individual egg clutches and thereafter the larval growth rate‐, survival‐ and size‐mediated priority effects under interspecific conditions. Eggs of both species hatched slightly faster at 24°C, and *S. vulgatum* eggs started hatching approximately 1 day earlier than *L. sponsa* eggs. However, this earlier hatching did not result in a size‐mediated priority effect, that is, a higher predation on the later hatching *L. sponsa*. Nevertheless, *L. sponsa* larvae were significantly larger than *S. vulgatum* at hatching. Growth rate and survival were significantly higher: (1) at 24°C compared with 21°C, (2) at high compared with low‐resource density and (3) in *L. sponsa* compared with *S. vulgatum*. Several significant interaction effects between resource density and temperature and between temperature and species were found. At high temperature, *L. sponsa* had a higher growth rate than *S. vulgatum*, but no difference in growth rate between species was found at low temperature. Additionally, a high‐resource density resulted in a higher growth rate in both species, but only under high temperature. There was a negative relationship between growth rate and survival in both species, suggesting that the higher growth rate of larvae was to some degree driven by intraguild predation and/or cannibalism. Our results imply that resource levels interact with temperature to affect interactions between the species.

## Graphical abstract

We tested how temperature and resource density affect survival and growth in the larval stage of two coexisting odonates, the damselfly *Lestes sponsa* and the dragonfly *Sympetrum vulgatum*. Our results imply that resource levels interact with temperature to affect interactions between the species. Theoretical studies should consider these effects when making predictions on changes in species distribution in light of climate change.

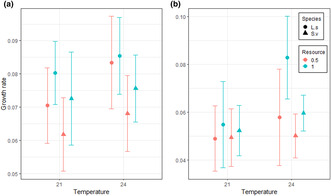

## 1 INTRODUCTION

Accumulating evidence suggests that climate change can affect interspecific species interactions (Suhling & Suhling, 2013; Tylianakis et al., 2008). These interaction effects result from various causes, one being the difference in temperature optima of species across several aspects, such as growth and development, metabolic rate and activity. Thus, a species that has a temperature optimum close to a predicted future temperature may have a competitive advantage over a species that has a temperature optimum close to the current temperature (Davis et al., 1998). A mechanistic understanding of changes in species interactions under climate change is, however, not straightforward. For example, Nilsson‐Örtman et al. (2014) showed that competitive interactions modified the temperature dependence of growth rates and survival, but that the strength and direction of these effects differed between species.

Along with temperature, many other variables can affect interactions between species. For a thorough understanding of how climate change might affect species interactions, it is important to explore how these other variables interact with temperature and in turn affect species interactions. One such variable that affects interspecific interactions is resource density (Hardin, 1960). The interaction between temperature and resource level has been explored theoretically using stoichiometric and ecosystem approaches that have focused on population and communities (Binzer et al., 2012; Cross et al., 2015; Dijoux et al., 2024). However, we know of no explicit theoretical models that have explored how the interaction between temperature and resources affects interspecific interactions using a mechanistic approach, but there could be several possible outcomes. For example, if increasing temperature has a negative effect on one of the two competing species, higher resource density might decrease this negative effect because more resources are available for the inferior competitor. In contrast, a higher resource level might also increase the negative effect if the superior competitor at the higher temperature if this species is also the superior competitor in terms of resource use. We do not know any empirical studies besides Rudolf (2022) that have examined how the interaction between temperature and resources affects interspecific competition. That study (Rudolf, 2022) showed that the effect of warming and resources may differ between predator–prey systems, such that nutrient addition enhanced the positive effect of early‐arrival species in one of the studied systems, whereas warming enhanced the negative effect of late‐arriving species in the other system (Rudolf, 2022).

Climate change also alters the phenology (seasonal life‐history events) of species, such as the emergence of insects, the arrival time of migrating birds and the flowering time of plants (Roslin et al., 2021). As species vary in their phenological responses to climate change, this variation may subsequently change the interspecific interaction among species in light of climate change (Roslin et al., 2021; Yang & Rudolf, 2010). Several studies have investigated such interaction effects, typically by manipulating life‐history events such as the hatching time of larvae (Rudolf & McCrory, 2018). By hatching earlier, one of the species can gain a size advantage, resulting in a positive effect on growth and survival compared with the species that hatch later (Rudolf & McCrory, 2018). Such size differences could be advantageous in exploitation and/or interference competition, but they could also result in higher intraguild predation (Padeffke & Suhling, 2003; Rudolf, 2022; Rudolf & McCrory, 2018). Studying such interactions is important for understanding climate change effects (Yang & Rudolf, 2010), but the combined effect of temperature and resource levels on such phenological shifts has not been explored in this context.

As outlined above, the interspecific interactions that may affect the survival and growth of interacting species at different temperatures and resource densities could be of several types. It could be resource competition, where species show different optimal temperatures in their resource utilisation efficiency (Nilsson‐Örtman et al., 2014). It could also be interference competition, where species physically prevent each other from accessing a resource. If allospecific aggressive behaviour is temperature‐dependent, interference competition might also be temperature‐dependent (Hoffacker et al., 2018). If two species that compete for the same food resource are predators and prey on each other, intraguild predation could occur. This risk of intraguild predation has been shown to be temperature‐dependent (Frances & McCauley, 2018; Winkler & Greve, 2004). Another factor that indirectly affects species interactions is cannibalism, which likewise has been shown to be temperature‐dependent (Sniegula et al., 2019). Since cannibalism results in more food per capita at the intra‐ and interspecific levels, it has the potential to affect interspecific interactions (Crumrine, 2010).

Mechanistic studies at the level of individuals on temperature and resource level interactions have rarely been performed in aquatic competition systems (Rudolf, 2022). The main aim of this study was to explore how the interaction of temperature and food resource levels affects survival and growth in two coexisting species inhabiting the littoral zone of lentic waters. We performed an experimental study using larvae of the damselfly (Zygoptera) *Lestes sponsa* and the dragonfly (Anisoptera) *Sympetrum vulgatum*. We first exposed individual egg clutches of these species at two different temperatures to explore how hatching time (phenology) changes the possible size advantage between species. After the eggs hatched, we raised larvae of the two species under interspecific conditions at two temperatures and two different resource levels and estimated growth and survival. We analysed our data with generalised linear models with resource, temperature and species as factors. Significant interaction terms in such an analysis will inform us about whether species respond in similar ways to changes in temperature and food resource.

## 2 MATERIALS AND METHODS

### 2.1 Study organisms

Odonata larvae increase in size by several magnitudes during ontogeny (Wissinger, 1988), making them a good model species to observe size‐mediated interspecific interaction effects. We used larvae of *Lestes sponsa* and *Sympetrum vulgatum* to study interspecific interactions in the aquatic stage. We chose these two species because they are among the most common Odonata species in northern Europe (Boudot & Kalkman, 2015; Dijkstra & Lewington, 2006). Odonata larvae are intermediate predators in aquatic communities. They prey on other invertebrate predators, and they are prey for fish predators (Corbet, 1999). *Lestes* and *Sympetrum* species co‐occur in the littoral zone throughout Europe, for example, Macan (1964), Johansson et al. (2010). In addition, intraguild predation and cannibalism are common in Odonata larvae (Crumrine, 2005; Johansson, 1993), and there is great overlap in diet use in larvae of Odonata species (Blois, 1985; Cerini et al., 2019; Corbet, 1999; Johansson, 1993; Johnson, 1985; Pritchard, 1964). However, we lack information on the extent of diet overlap and intraguild predation between our two focal species. Thus, we had no a priori predictions regarding whether one or the other species would be the stronger competitor. We simply wanted to examine how resources and temperature affected larval interspecific interactions in two common species that compete for the same food resource, aquatic invertebrates. In central Sweden, the main flight season of *L. sponsa* occurs in July–August and of *S. vulgatum* in July–mid‐October (Sahlén, 1996). In Northen Europe, both species have a 1‐year generation cycle with eggs overwintering and hatching in spring, followed by rapid larval development (Corbet, 1956a, 1999).

### 2.2 Experimental design

We performed two experiments. The purpose of the first experiment was to test (1) how two different temperatures, 21 and 24°C, affect the hatching rate of eggs, and (2) how the two different temperatures, 21 and 24°C, and resource levels (low and high food) affect interspecific interactions in larvae in terms of growth and survival of the two Odonata species. We note that the resource level in nature can be contingent on temperature. However, exploring this effect was beyond the scope of our experiment. The two temperatures used are within the range of natural temperatures in ponds where the eggs used in the experiments were collected. During the main growth season of the larvae, from June until September (Dijkstra & Lewington, 2006), the minimum and maximum water temperatures are 15.4 and 27.5°C, respectively (Holzmann et al., 2022): data from the pond where we collected *Lestes sponsa*, see below. The average for the whole growth season for ponds at this latitude is 17°C (Nilsson‐Örtman et al., 2012). The difference of 3°C between our treatments fits within the range of predicted increase in temperature within the next 100 years (IPCC, 2022).

*Lestes sponsa* and *S. vulgatum* eggs that are produced in the second half of the flight period develop until pre‐diapause in the late summer, thereafter enter a diapause stage when water becomes colder (autumn and winter) and finally hatch at the onset of higher temperatures in the spring (Corbet, 1999). Eggs of both species were collected on 20 August 2022 from two small ponds in the city of Uppsala, Sweden. The eggs of *S. vulgatum* were collected by dipping the abdomens of mated females in a small plastic cup filled with water. Upon dipping, the females released the eggs. Eggs of *L. sponsa* were collected by placing mated females into plastic cups lined with moist filter paper. After 2–3 days, *L. sponsa* females oviposited their eggs on the filter paper, and the eggs were then placed in 1‐L plastic boxes filled with water. We obtained egg clutches from three *S. vulgatum* and five *L. sponsa* females. Eggs were held in water at 20°C and a 14:10‐h light:dark regime for 4 weeks. Thereafter, the eggs were overwintered at 4°C in total darkness until used for the experiment. In our experiment, the overwintering period ended on 23 January 2023. On this date, the eggs were put into jars (6.3 cm in diameter × 7.5 cm in height) filled with 150 mL of conditioned water. The conditioned water was aerated non‐chlorinated tap water from a 50‐litre plastic barrel, in which 10‐g grass was added 1 year prior to it being used. The grass consisted of fresh clippings from nature, comprising a mixture of *Poa*, *Deschampsia* and *Festuca* species in unknown proportions.

To compare the hatching rate between the two species at the two different temperatures, the jars were placed in two tanks with water (145 × 55 cm, 15 cm deep and filled with water up to approx. 5.5 cm). One tank was kept at a constant temperature of 21°C, and the other at a constant temperature of 24°C by using immersion heaters. The egg clutches from each female were divided into two halves, and one‐half of each clutch was placed under one of the temperatures. Water temperature was checked and adjusted (when necessary) on a daily basis, which resulted in a mean of 21.14°C (+0.90 SD) and 24.18°C (+1.06 SD), respectively. The photoperiod at the beginning of the experiment was 14.5:9.5‐h light:dark, corresponding to the situation in Uppsala in mid‐April, and was adjusted weekly thereafter to simulate the progress of the season. Light was provided by led ramps (6400 K, 2200 lumen). The number of eggs hatching each day from each clutch and temperature was then counted daily between 24 and 27 January. After being counted, the larvae were moved to another jar. All jars were then removed from the water tanks to make space for the interpecific interaction experiment and kept at room temperature in the range of 18.4–21.3°C. On 30 January, the number of hatched individuals was counted again, which provided the final count for the timing of egg‐hatching estimates. We note that the last eggs that had not hatched by 27 January might biased our results on temperature effect on egg hatching.

On 27 January, the larvae from the egg clutches were randomly mixed, and the interspecific experiment was started. Five individuals of each species were introduced into one of 80 plastic boxes (10.8 × 10.8 cm, 10 cm deep) filled to 2/3 depth with conditioned water with five grass stem clips in each. The larval density corresponded to 500 larvae of each species per m^2^. Density in nature for dragonflies and damselflies ranges from 0.6 to 4500/m^2^ (Corbet, 1999a, p. 611). Forty of the boxes were kept at 21°C and 40 at 24°C. For each temperature treatment, half of the boxes were given a high‐resource treatment ratio of 10 mL of laboratory cultured brine shrimp nauplii (*Artemia* sp., Ocean Nutrition^R^) per day (average number of shrimps per mL = 234, range = 210–250, *n* = 5), corresponding to a density of 2340 brine shrimp per box. The other half was given a low‐resource treatment of 5 mL per day corresponding to 1170 brine shrimp per box. This low‐resource level corresponds to 117 *Artemia* sp. per larva and day. Past studies have shown that this low‐resource level is enough to rear Odonata larvae until emergence (Everling & Johansson, 2022; Sniegula et al., 2017). This resulted in a 2 temperatures × 2 resource levels experimental design with 20 replicates in each treatment combination. The experiment was run for 10 weeks. After 5 weeks, boxes and temperature treatments were shifted between the tanks to control for potential environmental effects in the laboratory, such as slight difference in light level, air pressure and air turbulence.

After 5 and 10 weeks, interspecific interaction differences between the different treatments, resource and temperature, were estimated based on survival and growth rate. Survival was estimated as the number of alive larvae. Metamorphosing individuals were included in the number of survivors and were also used for calculating the growth rate. The growth rate was estimated as the size difference between larval size at the start of the experiment and the size of each individual at Week 5 or 10 divided by the number of days until Weeks 5 and 10, respectively.

Since it was impossible to track individuals throughout the 10‐week experiment, larval size at the start of the experiment was estimated on 10 randomly chosen individuals. Larval size at Weeks 5 and 10 was measured in all surviving larvae. Larval size was estimated as the distance between the outer edge of the eyes, that is, head width. Head width is a good estimate of overall larval size in Odonata (Benke, 1970). Head width was measured in ImageJ v.1.54b (Rasband, 2012) from photographs taken in Petri dishes that had a reference scale at the bottom. The initial larval range size was 0.37–0.45 mm and 0.45–0.75 mm for *S. vulgatum* and *L. sponsa*, respectively.

In an additional experiment, we studied the potential importance of cannibalism and intraguild predation in the first experiment. This experiment was started on 1 February and lasted 14 days, and we used larvae from the same egg clutches as in the first experiment. The experiments were run in small plastic boxes (6.3 cm in diameter × 7.5 cm in height and filled with approximately 125 mL of conditioned water and 3 grass stem clips). There were three treatments: 2 *L*. *sponsa*; 2 *S*. *vulgatum*; and 1 *L*. *sponsa* and 1 *S. vulgatum* in one box. All individuals in a box were of similar age and size. Each treatment had 10 replicates. We fed these larvae 0.5 mL of cultured brine shrimp (average = 234, range = 210–250, *n* = 5) once a day. At the end of the experiment, we counted the number of live larvae. We checked survival once a week and found no dead larvae or parts of dead larvae, suggesting that all missing individuals had been preyed upon. We note that this experimental design differs from the first experiment and therefore results on predation are not directly comparable, but they provide evidence of predation events.

### 2.3 Statistical analysis

The treatment effect on survival was tested with a generalised linear mixed‐effects model with binomial distribution, with proportional survival per box as the response variable, temperature, resource density and species as fixed factors, and box as a random effect. All two‐way and the three‐way interactions between resource, temperature and species were included in the model. The effect of the treatments on growth rate was also tested with a generalised linear mixed‐effects model with a normal distribution (the residuals did not deviate from normality). The response variable was the mean box‐specific growth rate based on head width. The factors were temperature, resource density and species, and the box was a random effect. All two‐way and the three‐way interactions between resource, temperature and species were included in the model. Analyses on survival and growth rate were run separately for Weeks 5 and 10, and all interaction terms were included. To test whether there was an association between the survival and growth of larvae in each box, we ran a regression model with the total number of survivors in each box (both species) and the mean growth for each species in that box. All tests were run in R (4.1.2; R Core Team, 2022) using the package lme4, and R‐codes for models of survival and growth can be found as a supplemental file.

## 3 RESULTS

### 3.1 Hatching

**FIGURE 1:**
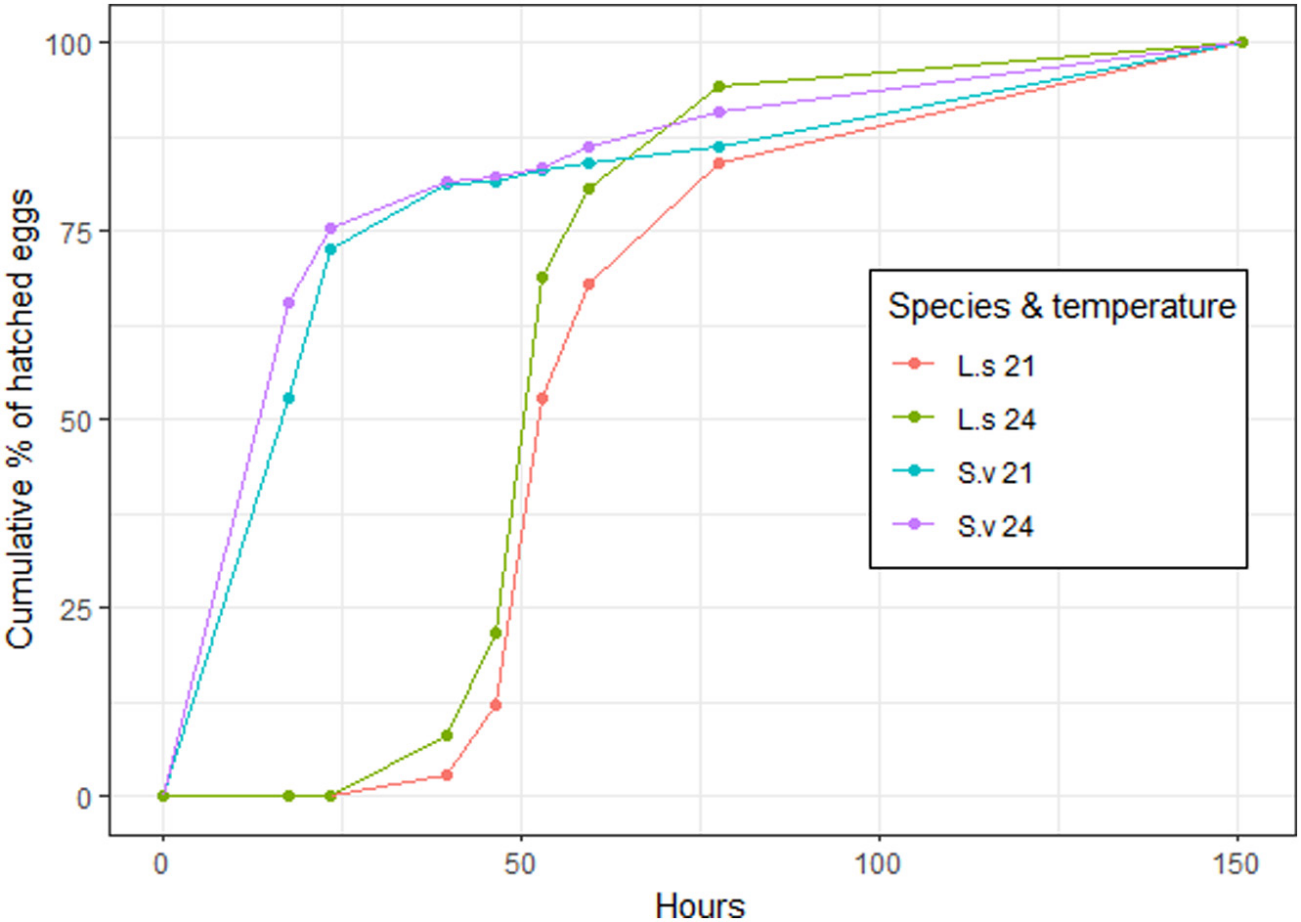
Cumulative percentage of eggs of *L. sponsa* (L.s) and *S. vulgatum* (S.v) kept at 21 and 24°C that hatched after 150 h. Dots represent the time points at which the number of hatched larvae was counted. L.s and S.v denotes *Lestes sponsa* and *Sympetrum vulgatum*, respectively.

After 6 days (150 h), 98% of the *L. sponsa* and 67% of the *S. vulgatum* eggs had hatched. No more eggs hatched after 6 days. We had too few clutch replicates for a statistical analysis, but visual inspection of the plot suggests that during the first 50 h of the hatching experiment, more eggs of both species had hatched at 24°C than at 21°C (Figure 1). In addition, initially, more *S. vulgatum* eggs had hatched, but after 75 h, the cumulative number of eggs that had hatched was similar for the two species (Figure 1). Note that between 27 January (after 75 h) and 30 January (after 150 h), the jars with the eggs were all held at room temperature (18.4–21.3°C). At hatching, *L. sponsa* larvae (mean = 0.56 mm) were significantly larger than *S. vulgatum* larvae (mean = 0.41 mm) (*t*‐test, *t* = 3.66, *p* = .002).

### 3.2 Survival

**FIGURE 2:**
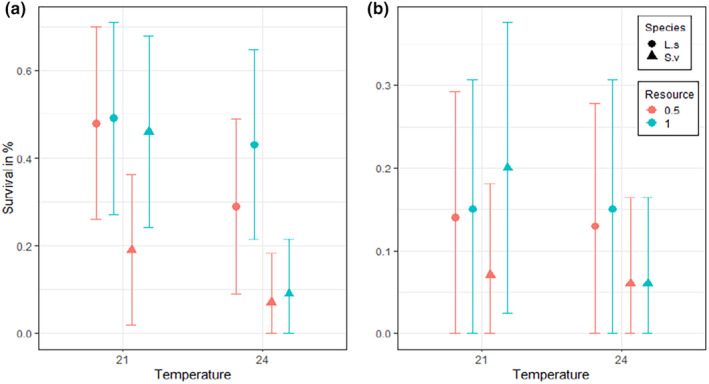
Larval survival (%) after 5 weeks (a) and 10 weeks (b) at 21 or 24°C and high or low‐resource density. Error bars represent the 95% confidence interval. L.s and S.v denotes *Lestes sponsa* and *Sympetrum vulgatum*, respectively. Resource is the amount of food given and 1 denotes a full ratio and 0.5 a half ratio, see methods for more details.

All three factors (species, resource density and temperature) had significant effects on survival at Week 5 (Table 1a). After 5 weeks, survival was on average higher at 21°C than at 24°C for both species. *L. sponsa* had a higher survival rate than *S. vulgatum*, and there was a positive effect of a higher resource level on survival (Figure 2a). There was also a significant interaction effect between resource density and species, temperature and species, and between resource density, temperature and species (Table 1a). These interaction effects were caused by several patterns. At 21°C, there was a difference in the survival of *S. vulgatum* depending on the resource density treatment, with individuals under a high‐resource level having a higher survival. At 24°C, however, the difference in survival between the resource levels was almost zero (Figure 2a), which could be due to the low survival in this treatment, resulting in a low sample size used for the analyses. In contrast, in *L. sponsa*, survival was higher under a high‐resource level at 24°C, but no difference was found between resource levels at 21°C.

**TABLE 1:** Effect of resource density, temperature and species on the survival of larvae after 5 weeks (a) and 10 weeks (b).

Variable	Chisq	df	Pr(>Chisq)
(a) Week 5			
Resource	9.4018	1	**.002**
Temperature	22.6802	1	**>.001**
Species	38.0103	1	**>.001**
Resource × Temperature	0.0546	1	.815
Resource × Species	4.2317	1	**.039**
Temperature × Species	10.7896	1	**.001**
Resource × Temp × Species	4.9051	1	**.026**
(b) Week 10			
Resource	2.5442	1	.110
Temperature	2.2967	1	.121
Species	3.3350	1	.068
Resource × Temperature	0.7060	1	.401
Resource × Species	1.5728	1	.210
Temperature × Species	2.8134	1	.093
Resource × Temp × Species	2.0494	1	.152

Bold values are significant.

After 10 weeks, very few individuals remained, and none of the factors or interaction terms was significant (Figure 2b, Table 1b). This non‐significant result might be a result of the low sample size used for the analyses.

### 3.3 Growth rate

**FIGURE 3:**
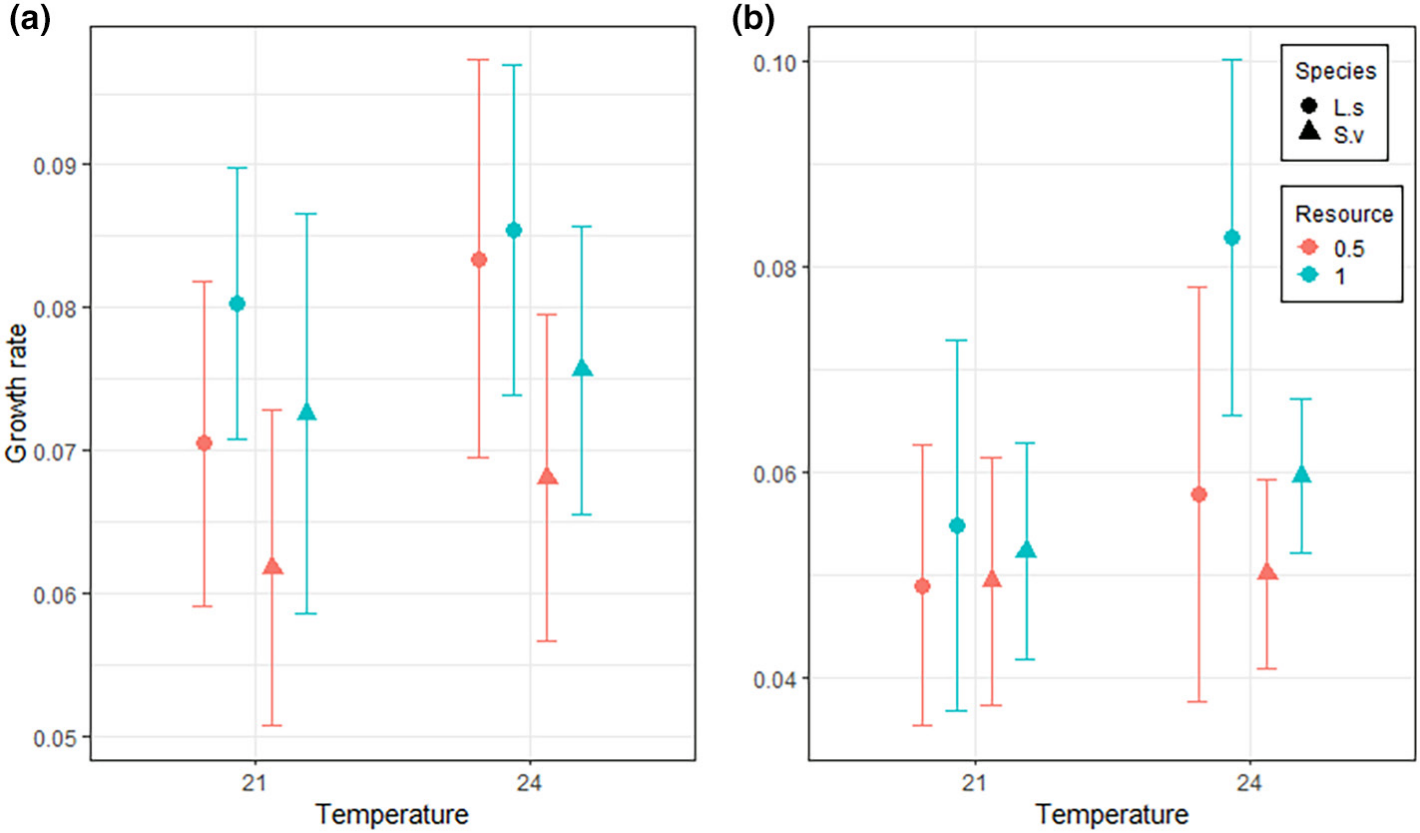
Growth rate (mm/day) of larvae after 5 weeks (a) and 10 weeks (b) at 21°C or 24°C and high‐ or low‐resource density. Error bars represent the standard deviation. L.s and S.v denotes *Lestes sponsa* and *Sympetrum vulgatum*, respectively. Resource is the amount of food given and 1 denotes a full ratio and 0.5 a half ratio, see methods for more details.

All three factors (species, resource density and temperature) had significant effects on the growth rate at Week 5 (Table 2a). After 5 weeks, the growth rate was on average higher for both species at 24°C and at the higher resource level (Figure 3a). The growth rate was generally higher for *L. sponsa* than for *S. vulgatum* (Figure 3a). There was a significant interaction effect between resource density and temperature, but no other interactions were significant (Table 2a). This interaction was caused by a higher growth rate at higher a resource levels at 21°C, but no difference in growth rate between resource levels at 24°C (Figure 3a).

**TABLE 2:** Effect of resource density, temperature and species on the growth rate of larvae after 5 weeks (a) and 10 weeks (b).

Variable	Chisq	df	Pr(>Chisq)
(a) Week 5			
Resource	23.6107	1	**<.001**
Temperature	25.2936	1	**<.001**
Species	28.2410	1	**<.001**
Resource × Temperature	5.0791	1	**.024**
Resource × Species	0.3401	1	.559
Temperature × Species	1.0236	1	.311
Resource × Temp × Species	0.2731	1	.601
(b) Week 10			
Resource	11.8578	1	**<.001**
Temperature	20.8887	1	**<.001**
Species	4.2848	1	**.038**
Resource × Temperature	6.8210	1	**.009**
Resource × Species	1.5600	1	.211
Temperature × Species	6.7799	1	**.009**
Resource × Tempe × Species	1.1520	1	.283

Bold values are significant.

All factors had a significant effect on the growth rate at Week 10, and there was a significant interaction effect between resource density and temperature and between temperature and species (Table 2b). After 10 weeks, the growth rate was on average higher at 24°C and at the higher resource level (Figure 3b). It was again also higher for *L. sponsa* than for *S. vulgatum* (Figure 3b). While the growth rate did not differ between resource levels at 21°C, the growth rate was higher at the high‐resource level at 24°C, and this pattern was observed in both species (Figure 3b). In addition, *L. sponsa* had a higher growth rate than *S. vulgatum* at 24°C but not at 21°C. (Figure 3b).

### 3.4 Correlation between survival and growth

Based on the survival counts and growth measurements in each box after 5 weeks, there was a significant negative relationship between survival and growth for *L. sponsa* in all treatments (Figure S1): at 21°C, low resource (*p* < .001, *R* ^2^ = .45); at 21°C, high resource (*p* = .001, *R* ^2^ = .40); at 24°C, low resource (*p* < .001, *R* ^2^ = .62); and at 24°C, high resource 1 (*p* < .001, *R* ^2^ = .56). For *S. vulgatum*, the relationship was only significant for one of the treatments: at 21°C, high resource (*p* = .05, *R* ^2^ = .16). The absence of a significant relationship was probably due to a very low sample size in *S. vulgatum* (Figure S1).

The data from the 10‐week final survival counts and growth measurements showed a significant negative relationship between growth and survival for *L. sponsa* at 24°C, low resource (*p* = .012, *R* ^2^ = .51), *S. vulgatum* at 21°C, low resource (*p* < .001, *R* ^2^ = .97) and *S. vulgatum* at 21°C, high resource (*p* < .001, *R* ^2^ = .67). For *L. sponsa*, a significant positive relationship between growth and survival was observed at 24°C, high resource (*p* = .036, *R* ^2^ = .37). There was no significant correlation for any of the other treatments (Figure S2).

### 3.5 Emergence

Approximately 5 weeks into the experiment, the first adults started to metamorphose: only individuals of *L. sponsa* emerged. Out of the 35 that had emerged at Week 10 (and could be traced back to the treatment: three were found outside the boxes and could not be traced back to the rearing boxes), 14 came from the 24°C + high‐resource treatment, 8 from the 24°C + low‐resource treatment, 7 from the 21°C + high‐resource treatment and 6 from the 21°C + low‐resource treatment.

### 3.6 Cannibalism and predation

In all of the jars of this experiment, at least one individual survived (Table S1). In the jars with two individuals of *S. vulgatum*, in 8 replicates out of 10 one individual ate the other. In the jars with two individuals of *L. sponsa*, one individual ate the other in 6 out of 10 replicates. In jars with one individual of each species, *S. vulgatum* ate *L. sponsa* in four out of 10 replicates, and *L. sponsa* ate *S. vulgatum* in 3 out of 10 replicates.

## 4 DISCUSSION

Few empirical studies have mechanistically examined how temperature and resource levels interact in shaping the effects of interspecific competition in aquatic systems. In contrast, several studies have used an ecosystem‐level approach focusing on energy and material flow (Binzer et al., 2012; Cross et al., 2015; Dijoux et al., 2024). Here, we found an interaction between temperature and resource density, and this interaction term differed between species and time in interesting ways. For example, at the end of the experiment, a high‐resource density resulted in a higher growth rate, but only at high temperature. Such interaction effects between temperature and resource density suggest that it is crucial to consider resource density in future models on how climate change can affect species interactions.

Temperature gradients at the microhabitat scale in the littoral zone of lakes and ponds are common (Andersen et al., 2017), and such gradients could potentially cause variation in egg hatching within and between species. We manipulated the temperature at the egg stage and found that *S. vulgatum* hatched approximately 1 day earlier than *L. sponsa*. However, at approximately 2.5 days, there was no obvious difference between the species in how many eggs had hatched. Since *S. vulgatum* hatched 1 day prior to *L. sponsa*, there was potential for a phenologically driven size‐mediated priority effect (Fincke, 1999; Padeffke & Suhling, 2003). However, we found no strong evidence for such an effect, since *L. sponsa* on average had a higher survival and growth rate than *S. vulgatum*. Nevertheless, this result does not exclude a priority effect. Additional experiments with and without a 1‐day priority design are needed to exclude such an effect.

We also found that *L. sponsa* had a higher growth and survival than *S. vulgatum*, and we suggest that this was mediated by an inherently higher growth rate in *L. sponsa* and higher predation by *L. sponsa* on *S. vulgatum*. In addition, since survival was lower for *S. vulgatum* than *L. sponsa*, this suggests that predation by *L. sponsa* on *S. vulgatum* resulted in a higher per capita food availability for *L. sponsa*. This last effect might also have contributed to the high growth in *L. sponsa*. We note, however, that our second experiment on cannibalism and intraguild predation did not show a higher predation on *S. vulgatum* by *L. sponsa* compared with the predation effects that occurred for either species separately.

### 4.1 Temperature

On average, survival was lower at 24°C than 21°C. We suggest that this was caused by a higher predation and cannibalism at 24°C. Several studies have shown that predation and cannibalism increase with temperature as long as the temperature remains below the optimal temperature for growth and survival (Öhlund et al., 2015; Sniegula et al., 2017, 2019; Walker et al., 2020). The mechanistic explanation is that activity is higher at higher temperatures, which in turn increases encounter rates, resulting in higher cannibalism and predation. The optimal temperature for the growth and survival of many aquatic insect larvae and odonate larvae is a couple of degrees above 24°C (Gallegos‐Sanchez et al., 2022; Suhling et al., 2015). Hence, our mechanistic explanation seems reasonable, but experimental evidence is needed to support our explanation for this particular study system. Several alternative but not mutually exclusive explanation for the lower survival at 24°C are possible. It could be that a higher growth at 24°C results in a higher intrinsic mortality caused by developmental instability and/or physiological costs (Arendt, 1997; Gotthard, 2001). In addition, larvae might be more food limited due to higher metabolic costs at higher temperature, which could cause a higher mortality (Sentis et al., 2015).

The growth rates we found correspond to those estimated in other studies: between 0.04 and 0.05 mm/day for *S. vulgatum* (Hogreve & Suhling, 2022) and between 0.07 and 0.08 mm/day for *L. sponsa* (Sniegula et al., 2014). Hence, the growth rate we found does not seem to be biased by our experimental design. Overall, the growth rate was higher at 24°C, agreeing with previous results showing that the thermal performance curves for Odonata larvae in general have a maximum at approximately 27°C (Suhling et al., 2015). More specifically, Suhling et al. (2015) estimated the optimal growth rate temperature to be 26.6°C for *S. vulgatum* and 24.8°C for congeneric species *Lestes disjunctus* (Krishnaraj & Pritchard, 1995). However, the growth rate in our experiment was also affected by mortality. As mortality increases, there will be more food per capita, which results in higher growth. We will discuss this below.

### 4.2 Resource density

Survival and growth were on average higher at high‐resource levels. Higher survival effects at higher resource levels have also been found in prey in an aquatic predatory–prey system, but this effect decreased with later arrival time for the prey (Rudolf, 2022). The higher survival at the high‐resource level was probably an effect of milder intra‐ and interspecific interactions. At high‐resource density, individuals reach a higher level of satiation, which usually results in less intraguild predation and cannibalism (Johansson, 1992, 1993). It is probably less risky to prey on *Artemia* compared with preying on conspecifics or other members of the predation guild. In addition, at higher resource levels, foraging activity is reduced (Anholt & Werner, 1995), which probably results in lower mortality at high‐resource levels.

### 4.3 Interaction between temperature and resource density

We found several interactions between resource density and temperature. Such interaction could be of several kinds (Rudolf, 2022). For example, if increasing temperature has a negative effect on one of the competing species in a species‐pair competition system, a higher resource level might decrease this negative effect because more resources are available for the inferior competitor (Anholt & Werner, 1995). However, a higher resource level might also increase the negative effect if the superior competitor at the higher temperature is also the superior competitor in terms of resource use (Rudolf & McCrory, 2018). Here, we found few such effects on growth since none of the three‐way interactions (species × resource density × temperature) was significant. However, for mortality at 5 weeks, we found such an effect, suggesting that increasing resources increased the negative effect of *L. sponsa* on S. *vulgatum* at 24°C. However, after 10 weeks, the pattern was no longer present, which was probably due to the few surviving individuals left in the boxes.

*Lestes sponsa* was the superior competitor since it had a higher growth rate and a lower mortality compared with *S. vulgatum*. In addition, all the emerged individuals were *L. sponsa*, and the highest number of emergences was observed at 24°C and high‐resource level. This emergence result is also supported by the higher growth rate in this treatment. This higher success in terms of growth and survival was not driven by a phenological priority effect since *L. sponsa* did not hatch before *S. vulgatum*. However, *L. sponsa* had a slightly larger initial size at hatching, that is, at the start of the experiment, suggesting that a size‐mediated initial body size effect might be possible. We suggest that the higher growth rate and lower mortality of *L. sponsa* were driven by a combination of a size priority effect, higher foraging activity, higher intrinsic growth rate and higher aggressive behaviour resulting in higher predation on *S. vulgatum*. We found some support of these suggestions, since *L. sponsa* had a higher growth rate compared with *S. vulgatum* in some of our treatments. *L. sponsa* also had a larger size at hatching compared with *S. vulgatum*, suggesting a size priority effect. The higher growth of *L. sponsa* compare with *S. vulgatum* corresponds to what has been found in other studies (e.g., Hogreve & Suhling, 2022; Sniegula et al., 2014). However, Johansson (2000) found that another *Sympetrum* species, *S*. *danae*, had a higher activity than *L. sponsa*. These suggestions are speculative and mechanistic studies are needed to confirm which of the variables contribute the most to the success of *L. sponsa*.

Interestingly, this effect on mortality and growth rate differed between weeks, resource levels and temperatures. While mortality was lower for *L. sponsa* compared with *S. vulgatum* at both resource levels at 24°C, it was only lower at low‐resource levels at 21°C, resulting in species × temperature and species × resource × temperature interactions. One possible mechanism for the lower survival of *S. vulgatum* at the low‐resource level at 21°C may be the higher activity of *L. sponsa* compared with *S. vulgatum* at this temperature. At low‐resource levels, *L. sponsa* probably searches more actively for prey, resulting in more encounters and higher predation on *S. vulgatum*. Higher activity level at low‐resource level has been occurred in many species (Anholt & Werner, 1995; Johansson, 1992). At Week 10, there were very few significant effects on mortality. This was presumably due to the low number of larvae still alive at this time, which reduces the statistical power. Although we tried to mimic natural larval densities, the low number of live larvae was probably due to a very high rate of predation and cannibalism, which was most likely caused by edge effects in the boxes (i.e., when animals gather along the edges of the plastic boxes in our experiment) during the 10 weeks of the experiment (Bergström & Englund, 2002).

For growth rate, the pattern of significant interactions was different compared with those for survival, and there were significant interaction effects after 5 and 10 weeks. The growth rate of *L. sponsa* was higher than that of *S. vulgatum*, and this effect was temperature‐ and resource‐dependent. First, at Week 5, the growth rate was relatively higher for *L. sponsa* than for *S. vulgatum* at 24°C. This difference could have been driven by the lower number of *S. vulgatum* that survived at this temperature in Week 5, which would leave more food resources for *L. sponsa*. Second, while a higher resource level resulted in a higher growth rate *in L. sponsa* and *S. vulgatum* at 21°C after 5 weeks, there was no such effect at 24°C. One interpretation of the second result could be that at 21°C after 5 weeks, all *L. sponsa* had not grown enough to be able to eat other larvae, resulting in higher survival and higher competition for resources (Anholt, 1994). Since the growth rate was higher at 24°C, individuals at 24°C had grown more and were all more or less able to eat other larvae, resulting in fewer individuals, less competition and therefore resources being less important. Third, in contrast to Week 5, there was no difference in the growth rate at 21°C at Week 10. However, there was a higher growth rate for *L. sponsa* at 24°C, but no such effect was observed on *S. vulgatum*. In addition, the growth rate was higher at high‐resource levels. There could be many mechanistic explanations for these last interaction terms. For example, after 10 weeks at 24°C, many of the larvae had already been eaten or died, and resources became important again since they were the only source of food.

In summary, more detailed mechanistic observations on behaviour and actual observations of whether mortality was caused by intraguild predation or cannibalism are needed for a better understanding. Nevertheless, our results suggest that resource levels interact with temperature and cause differences in the strength of interactions between species.

### 4.4 Correlation between survival and growth

There was a significant negative correlation between survival and growth for both species in several of the treatments. This is expected since per capita food increases with fewer competitors, and consequently, more energy for growth is available. This correlation is also commonly seen in similar experiments (Everling & Johansson, 2022; Van Buskirk, 1993). Interestingly, at Week 10, the correlation was positive for *L. sponsa* at 24°C at the high‐resource level. This could be because at Week 10, there were very few survivors left, but the ones that had survived were those with a high growth rate, which was probably caused by high food per capita.

### 4.5 Predation effects

We could not determine the cause of death of the larvae in the experiment. For example, did they die from predation, starvation or physiological stress? Very few dead larvae were found (Table S3). However, the additional predation and cannibalism experiment we ran did show both cannibalism and intraguild predation. In addition, in the 10‐week experiment, parts of dead bodies were found, indicating that the dead larva had been eaten. Predation in Odonata is a common phenomenon (Corbet, 1999; Raczyński et al., 2021; Wissinger, 1989), and it has been previously shown that size asymmetry specifically often leads to predation between Odonata larvae (Rasmussen et al., 2014; Suhling & Suhling, 2013; Wissinger, 1989). Hence, we suggest that most of the mortality that occurred was due to interspecific predation or cannibalism.

### 4.6 Implications in nature

Since a higher temperature increases the competitive ability of *L. sponsa*, this might shift the competitive balance between *L. sponsa* and *S. vulgatum* and perhaps also between other species in the aquatic food web that are connected with *L. sponsa* as prey or predators. Several studies have found that *Lestes* and *Sympetrum* co‐occur in the same microhabitat (Johansson et al., 2010; Johansson & Brodin, 2003; Macan, 1964). Since *L. sponsa* usually emerges before *S. vulgatum* in natural environments (Valle, 1938), the effects seen in the experiments could be even stronger in nature. Large differences in arrival between Odonata larvae have been suggested to possibly lead to exclusion from habitats for late comers (Rudolf & McCrory, 2018). However, it should be noted that the predation effects in our experiment might be higher than those in nature because of the higher encounter rate resulting from the confined space in the boxes (Costa et al., 2022; Van Buskirk, 1989). The larval development time of ca. 2 months found in the literature (Corbet, 1956b) could perhaps also change in a scenario of higher temperatures, and such effects have been found in other studies (Rudolf, 2022). In general, larval development time in insects declines with increasing temperature, the degree‐days concept (Baskerville & Emin, 1969). Rudolf and McCrory (2018) suggest that priority effects could be more prominent in productive environments. Considering that higher temperature and higher production often go hand‐in‐hand and that higher temperature itself can influence priority effects, the competitive relations of species in such habitats are likely important to focus on in ecological predictions of climate change. To investigate this further, it would be informative to look at populations in a field experiment including the complete food web available and manipulating temperature.

## AUTHOR CONTRIBUTIONS

**Frank Johansson:** Conceptualization (equal); data curation (equal); formal analysis (supporting); funding acquisition (lead); investigation (equal); methodology (equal); project administration (supporting); resources (equal); validation (equal); writing – original draft (equal); writing – review and editing (lead). **Lisa Stahl:** Conceptualization (supporting); data curation (equal); formal analysis (lead); funding acquisition (supporting); investigation (equal); methodology (equal); project administration (supporting); resources (equal); validation (equal); visualization (equal); writing – original draft (equal); writing – review and editing (supporting).

## CONFLICT OF INTEREST STATEMENT

The authors have no conflicts of interest.

## Supporting information


Data S1.

Data S2.

## Data Availability

The data that support the findings of this study are available as Excel files in Supporting Information of this article.
