# Supplementary material for: Effects of temperature and resource level on interspecific interactions in two species of Odonata larvae
Source: Ecol Evol. 2024 Jun 13;14(6):e11502. doi: 10.1002/ece3.11502 (PMC11170025; doi:10.1002/ece3.11502)
Supplement: Supplementary file 2 — Data S2. [file ECE3-14-e11502-s002.zip › Supplements Figures and Tables Johansson and Stahl.docx]

**Supplements: Effects of temperature and resource level on interspecific interactions in two species of Odonata larvae**

**Lisa Stahl and Frank Johansson**


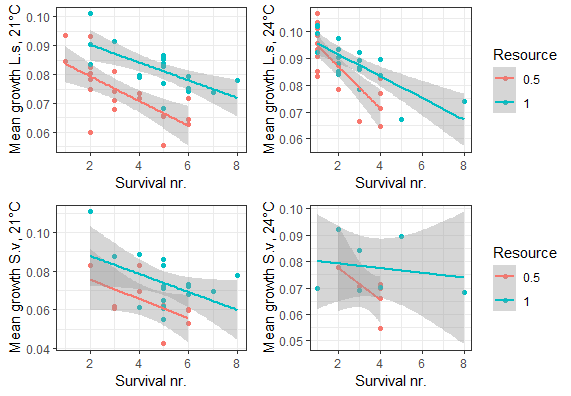


Figure S1. Correlation between growth and survival for the two species in the different treatments after 5 weeks.


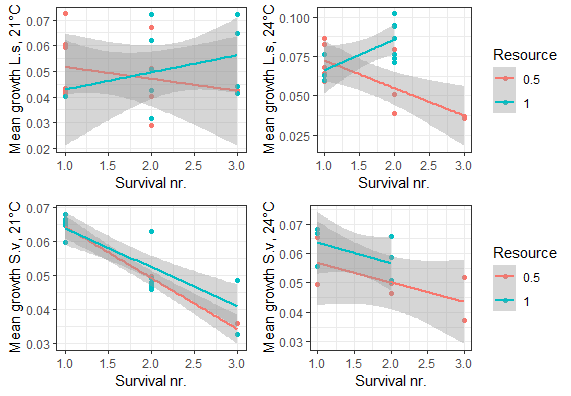


Figure S2. Correlation between growth and survival for the two species in the different treatments after 10 weeks.

| Treatment | Alive | Dead | Not found |
| --- | --- | --- | --- |
| L.s, 21, low | 2.4 | 0.35 | 2.25 |
| L.s, 21, high | 2.45 | 0.2 | 2.1 |
| L.s, 24, low | 1.45 | 0.05 | 3.4 |
| L.s, 24, high | 2.15 | 0.05 | 2.8 |
| S.v, 21, low | 0.95 | 0.05 | 4 |
| S.v, 21, high | 2.3 | 0.05 | 2.55 |
| S.v, 24, low | 0.25 | 0.05 | 4.45 |
| S.v, 24, high | 0.45 | 0 | 4.55 |

Table T1. Mean number of individuals per box that were found alive, dead or were not found after 5 weeks. Results are grouped per treatment. "Not found" indicates that neither corpses nor living larvae were discovered.
